# Germacranolides from *Carpesium divaricatum*: Some New Data on Cytotoxic and Anti-Inflammatory Activity

**DOI:** 10.3390/molecules26154644

**Published:** 2021-07-30

**Authors:** Natalia Kłeczek, Janusz Malarz, Barbara Gierlikowska, Łukasz Skalniak, Agnieszka Galanty, Anna K. Kiss, Anna Stojakowska

**Affiliations:** 1 Maj Institute of Pharmacology, Polish Academy of Sciences, Smętna 12, 31-343 Kraków, Poland; kleczek@if-pan.krakow.pl (N.K.); malarzj@if-pan.krakow.pl (J.M.); 2 Department of Laboratory Diagnostics and Clinical Immunology of Developmental Age, Medical University of Warsaw, Żwirki i Wigury 63a, 02-091 Warsaw, Poland; barbara.gierlikowska@wum.edu.pl; 3 Faculty of Chemistry, Jagiellonian University, Gronostajowa 2, 30-387 Kraków, Poland; lukasz.skalniak@uj.edu.pl; 4 Department of Pharmacognosy, Jagiellonian University Medical College, Medyczna 9, 30-688 Kraków, Poland; mfgalant@cyf-kr.edu.pl; 5 Department of Pharmacognosy and Molecular Basis of Phytotherapy, Medical University of Warsaw, Banacha 1, 02-097 Warsaw, Poland; akiss@wum.edu.pl

**Keywords:** anti-inflammatory, *Carpesium*, cytokines, cytotoxicity, germacranolides, Inuleae, p53, ROS, sesquiterpene lactones

## Abstract

*Carpesium divaricatum* Sieb. & Zucc., a traditional medicinal plant used as an inflammation-relieving remedy, is a rich source of terpenoids. At least 40 germacrane-type sesquiterpene lactones, representatives of four different structural groups, were isolated from the plant. Cytotoxicity against cancer cells in vitro is the most frequently described biological activity of the compounds. However, little is known about the selectivity of the cytotoxic effect. The anti-inflammatory activity of the germacranolides is also poorly documented. The objective of the present study was to assess the cytotoxic activity of selected *C. divaricatum* germacranolides-derivatives of 4,5,8,9-tetrahydroxy-3-oxo-germacran-6,12-olide towards cancer and normal cell lines (including cells of different p53 status). Moreover, to assess the anti-inflammatory effect of the compounds, the release of four proinflammatory cytokines/chemokines (IL-1β, IL-8, TNF-α and CCL2) by lipopolysaccharide-stimulated human neutrophils was measured by ELISA. The investigated sesquiterpene lactones demonstrated nonselective activity towards prostate cancer (Du145 and PC3) and normal prostate epithelial cells (PNT2) as well as against melanoma cells (A375 and HTB140) and keratinocytes (HaCaT). Cytotoxic activity against osteosarcoma cells was independent of their p53 status. In sub-cytotoxic concentrations (0.5–2.5 µM) the studied compounds significantly decreased cytokine/chemokine release by lipopolysaccharide-stimulated human leukocytes.

## 1. Introduction

Plants from the genus *Carpesium* (Asteraceae, Inuleae, Inulineae), native to Asia and Europe, have long been used by the traditional systems of medicine in Far Eastern countries. Preparations of whole *Carpesium* plants have been applied as antipyretic, analgesic, antiparasitic and inflammation-relieving remedies. In China, Korea and Japan, *C. divaricatum* Sieb. & Zucc. (Jin Wa Er) found use in the treatment of common cold, fevers, toothache, sore throat, parasitic infestations, diarrhea and urinary tract infections [1,2].

Phytochemical studies on *C. divaricatum* started in the 1990s with the isolation of three germacranolide-type sesquiterpene lactones by Maruyama [3]. Since that time, over 40 compounds from this class of secondary metabolites have been isolated from plants of the species [4,5,6,7,8,9]. Moreover, acyclic diterpenes, monoterpenoid thymol derivatives, 12-oxo-phytodienoic acid and numerous caffeic acid derivatives were identified as constituents of *C. divaricatum* [10,11,12]. The composition of essential oils distilled from the plant roots and aerial parts was also described [13].

Germacranolides of *C. divaricatum*, based on their structural features, can be divided into four groups: (I) derivatives of 2β,5-epoxy-5β,6,9,10-tetrahydroxy-germacran-8,12-olide, (II) derivatives of 2α,5-epoxy-5α,6,9,10-tetrahydroxy-germacran-8,12-olide, (III) derivatives of 4,5,8-trihydroxy-9-oxo-germacran-6,12-olide and (IV) derivatives of 4,5,8,9-tetrahydroxy-3-oxo-germacran-6,12-olide [9]. The majority of the compounds demonstrated cytotoxic activity towards human cancer cell lines in vitro [5,6,7,8,9]. The selectivity of this effect, however, has not been studied. Cardivarolide H (group IV) and incaspitolide A (group III) appeared to induce apoptosis in HepG2 and PC3 cells, respectively [9,14]. Incaspitolide A exerted its effect via inhibition of the PI3K/Akt/xIAP pathway and upregulation of p53 expression [14].

The anti-inflammatory activity of sesquiterpene lactones was extensively studied in the late 1990s and early 2000s [15,16,17,18]. The research revealed the role of the compounds as inhibitors of the NF-κB signaling pathway. The transcription factor NF-κB, which is a key regulator of the inflammatory process, is also implicated in the promotion of tumor cells’ proliferation and suppression of apoptosis [19]. Studies on structure–cytotoxicity relationships of sesquiterpene lactones (e.g., [20,21]) and structure–activity relationship of sesquiterpene lactones as inhibitors of the transcription factor NF-κB (e.g., [18,22]) have been recently summarized by Schmidt [23]. As both cytotoxic and anti-inflammatory activities of the compounds are dependent on the presence of α,β-unsaturated carbonyl functional groups (including α-methylene-γ-lactone) and are based on the same chemical phenomenon (Michael addition to the respective structural fragments of macromolecules), they are very unlikely to occur separately. This is important when assessing benefit–risk balance for sesquiterpene lactone containing plant preparations.

The NF-κB inhibitory effect was shown for two lactones isolated from *C. divaricatum*: 2β,5-epoxy-5,10-dihydroxy-6α-angeloyloxy-9β-isobutyryloxy-germacran-8α,12-olide (structural group I, concentration of 10 μM) and 2α,5-epoxy-5,10-dihydroxy- 6α-angeloyloxy-9β-(3-methylbutyryloxy)-germacran-8α,12-olide (group II, concentration range: 1–10 μM) [24,25]. Both compounds demonstrated cytotoxic activity against cancer cells in vitro (IC_50_ > 7.9 μM and IC_50_ > 7.6 μM, respectively) [8,26].

The objective of the present study was to assess the anti-inflammatory activity of the yet unexplored group IV of *C. divaricatum* germacranolides and to evaluate the selectivity of cytotoxic effects exerted by the compounds.

## 2. Results

### 2.1. Identification of Isolated Sesquiterpene Lactones

**Figure 1:**
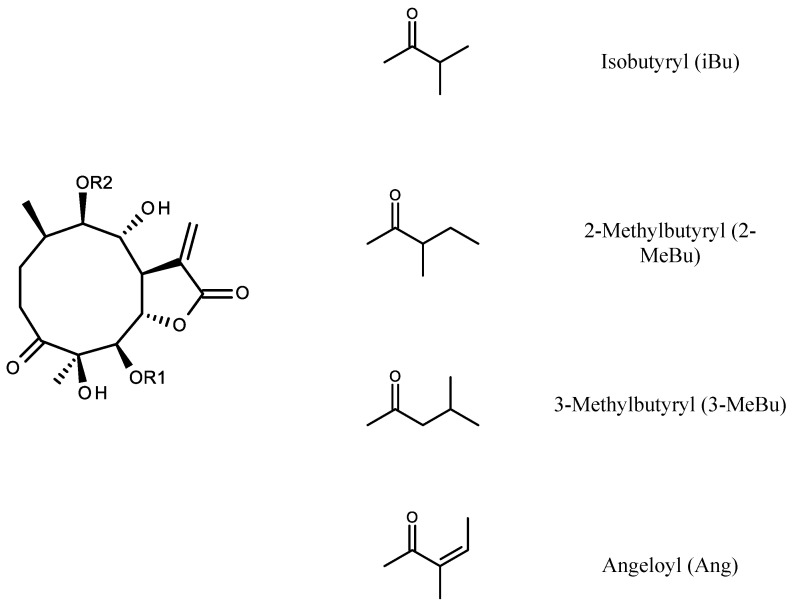
Chemical structures of 4β,8α-dihydroxy-5β-angeloyloxy-9β-(2-methylbutyryloxy)-3-oxo-germacran-6α,12-olide (**1,** R1 = Ang, R2 = 2-MeBu), 4β,8α-dihydroxy-5β-angeloyloxy-9β-(3-methylbutyryloxy)-3-oxo-germacran-6α,12-olide (**2,** R1 = Ang, R2 = 3-MeBu) and 4β,8α-dihydroxy-5β-isobutyryloxy-9β-(3-methylbutyryloxy)-3-oxo-germacran-6α,12-olide (**3,** R1 = iBu, R2 = 3-Mebu).

Chromatographic separation of the chloroform extract from aerial parts of *C. divaricatum* gave several fractions rich in sesquiterpene lactones. From the fractions, a few germacranolides were isolated in high yield and purity. Out of them, three structurally related compounds (**1**–**3**) were chosen to perform in vitro assays. The compounds were identified on the basis of their spectroscopic data as 4β,8α-dihydroxy- 5β-angeloyloxy-9β-(2-methylbutyryloxy)-3-oxo-germacran-6α,12-olide (**1**) [8,27], cardivarolide G (4β,8α-dihydroxy-5β-angeloyloxy-9β-(3-methylbutyryloxy)-3-oxo-germacran- 6α,12-olide, **2**) [8] and 4β,8α-dihydroxy-5β-isobutyryloxy-9β-(3-methylbutyryloxy)- 3-oxo-germacran-6α,12-olide (**3**) [8,28] (see Figure 1).

### 2.2. Cytotoxic Activities of ***1***–***3*** against Selected Cancer Cell Lines

#### 2.2.1. Viability of Cancer and Normal Cell Lines Treated with **3**

Normal prostate epithelial cells (PNT2), two prostate cancer cell lines (Du145 and PC3), human keratinocytes (HaCaT), two melanoma cell lines (A375 and HTB140) and two colon cancer cell lines (HT29 and Caco-2) were treated with **3** at a concentration range 1.0–50.0 µg/mL. Viability of the cells was estimated using lactate dehydrogenase (LDH) assay. Results of the experiment are summarized in Table 1.

The examined compound demonstrated moderate cytotoxicity to both cancer and normal cells in vitro. It is worth noting that **3** was more active against PC3 cells than doxorubicin, the reference cytostatic drug. IC_50_ values of **3** ranged from 3.88 µg/mL (Caco-2 cells) to 16.39 µg/mL (PNT2 cells). All cell lines used in the experiment were susceptible to the studied compound.

#### 2.2.2. Effects of **1** and **2** on Two Lines of Osteosarcoma with Different p53 Status

**Figure 2:**
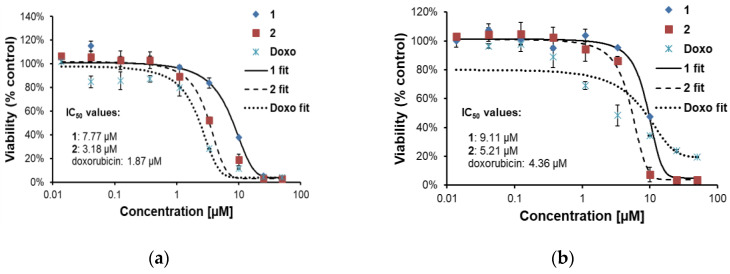
Viabilities of osteosarcoma cell lines U-2 OS (wild-type p53 status) and SAOS-2 (with p53 deletion) after 24 h treatment with **1**, **2** and doxorubicin (Doxo): (**a**) U-2 OS cells; (**b**) SAOS-2 cells.

**Figure 3:**
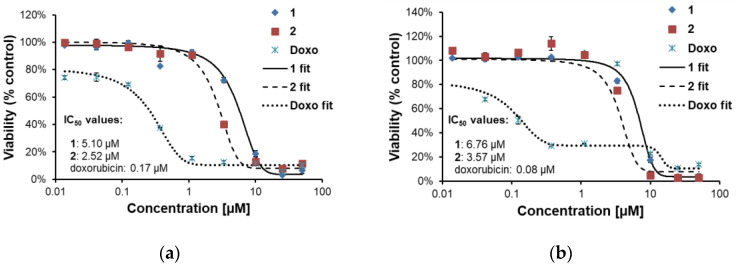
Viabilities of osteosarcoma cell lines U-2 OS (wild-type p53 status) and SAOS-2 (with p53 deletion) after 48 h treatment with **1**, **2** and doxorubicin (Doxo): (**a**) U-2 OS cells; (**b**) SAOS-2 cells.

Two osteosarcoma cell lines: U-2 OS (with wild-type p53 status) and SAOS-2 (p53 null cell line) were used to evaluate cytotoxic activity of **1** and **2**. Doxorubicin was employed as a reference drug. Viability of the cells treated by **1** or **2** (concentration range 0.014–50.0 µM) for 24 and 48 h was assessed by thiazolyl blue tetrazolium bromide (MTT) test. Results are shown in Figure 2 and Figure 3.

### 2.3. Effect of ***1*** and ***2*** on Lipopolysaccharide (LPS)-Stimulated Release of Proinflammatory Cytokines from Human Neutrophils

#### 2.3.1. Cytotoxicity

**Figure 4:**
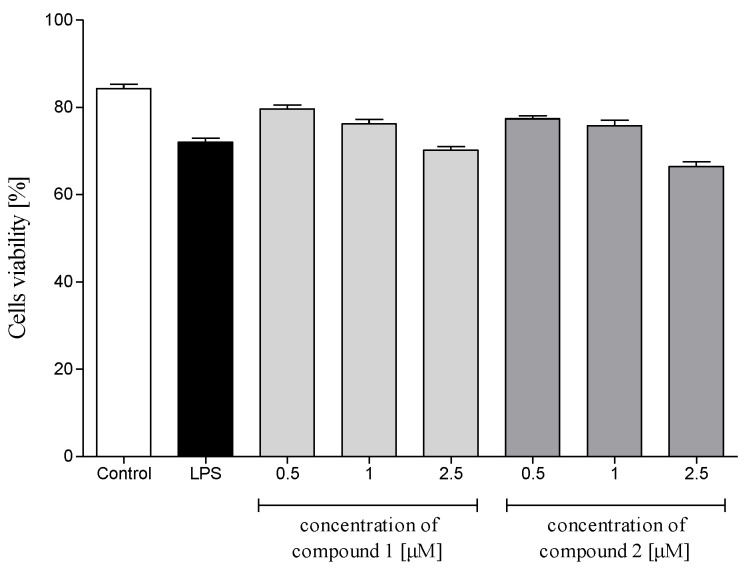
Cytotoxic effects of **1** (4β,8α-dihydroxy-5β-angeloyloxy-9β-(2-methylbutyryloxy)-3-oxo-germacran-6α,12-olide) and **2** (cardivarolide G), at concentrations 0.5, 1.0 and 2.5 μM, on human LPS-stimulated neutrophils. Results shown as percentage of cells without diminished membrane integrity (propidium iodide negative cells). Control, untreated cells; LPS, cells stimulated with LPS (stimulated control). Statistical significance: * *p* < 0.05, with reference to a stimulated control.

Cytotoxicity of **1** and **2** to human polymorphonuclear leukocytes (PMNs) was evaluated using propidium iodide (PI) staining and flow cytometry (FACS) analysis. The compounds were nontoxic in PMNs at 2.5 µM or lower concentrations (Figure 4). Solvent (DMSO) did not affect viability of the cells (data not shown). On the basis of these results, all further experiments were performed using the investigated sesquiterpene lactones at concentrations up to 2.5 µM.

#### 2.3.2. Reactive Oxygen Species (ROS) Production

**Figure 5:**
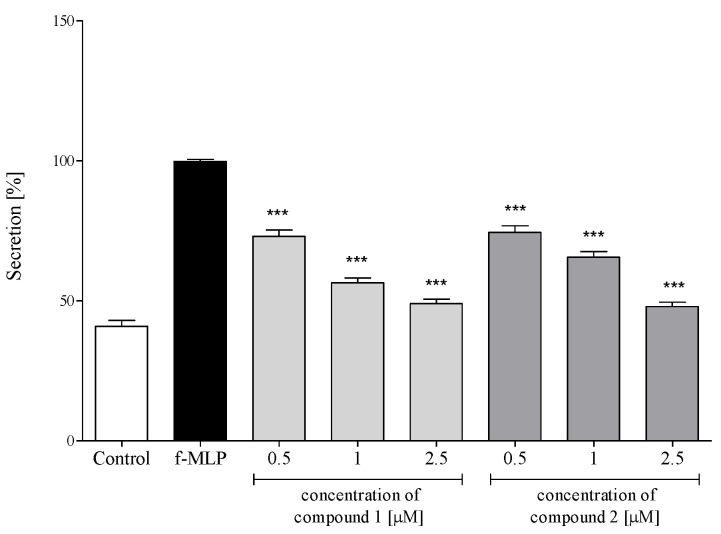
Inhibitory effects of **1** (4β,8α-dihydroxy-5β-angeloyloxy-9β-(2-methylbutyryloxy)-3-oxo-germacran-6α,12-olide) and **2** (cardivarolide G), at concentrations 0.5, 1.0 and 2.5 μM, on the ROS release from f-MLP-stimulated human neutrophils. Statistical significance: *** *p* < 0.001, with reference to a stimulated control.

Activation of PMNs, either at a site of inflammation or induced in vitro by proinflammatory agents, results in an oxidative burst in these cells. The phenomenon is characterized by intense ROS generation and liberation of proteolytic enzymes from azurophilic granules. An effect of the sesquiterpene lactones **1** and **2** on ROS production in PMNs was assessed in response to N-formyl-Met-Leu-Phe (f-MLP) stimulation. The examined sesquiterpenoids significantly and dose-dependently reduced ROS generation at a concentration range of 1–2.5 μM (Figure 5).

#### 2.3.3. Release of Selected Proinflammatory Cytokines/Chemokines (IL-1β, IL-8, TNF-α, CCL2)

**Figure 6:**
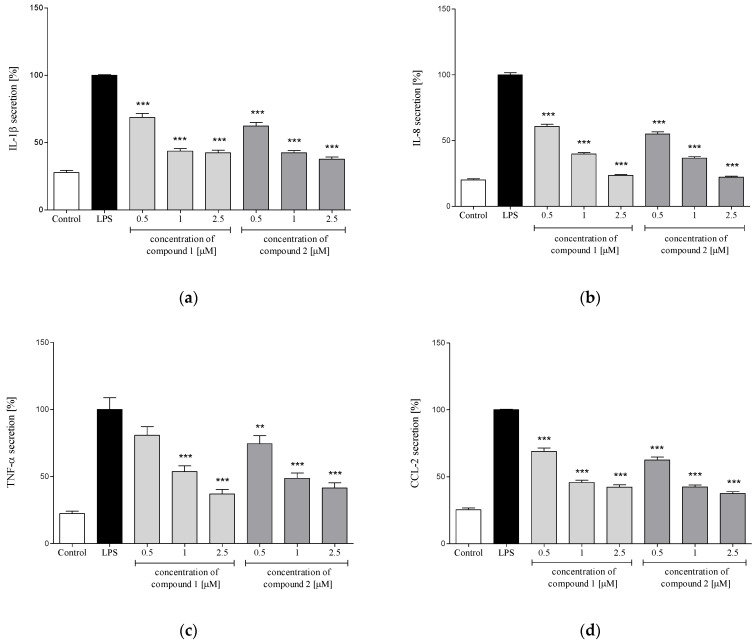
Inhibitory effects of **1** (4β,8α-dihydroxy-5β-angeloyloxy-9β-(2-methylbutyryloxy)-3-oxo-germacran-6α,12-olide) and **2** (cardivarolide G), at concentrations 0.5, 1.0 and 2.5 μM, on IL-1β (**a**), IL-8 (**b**), TNF-α (**c**) and CCL2 (**d**) secretion by LPS-stimulated human neutrophils. Statistical significance: ** *p* < 0.01, *** *p* < 0.001, with reference to a stimulated control.

In response to stimulation with proinflammatory agents, e.g., LPS or f-MLP, human neutrophils excrete an array of cytokines and chemokines, including TNF-α, IL-1β, IL-8 and CCL2 [29,30]. In the present study, neutrophils were pretreated with the examined compounds (**1** or **2**) before their priming with LPS. Levels of IL-1β, IL-8, TNF-α and CCL2 were determined in the culture medium, using ELISA, 24 h after LPS stimulation. Preincubation of human neutrophils with each of the investigated compounds (at concentrations of 0.5, 1.0 and 2.5 µM) caused significant and dose-dependent inhibition of IL-1β, IL-8 and CCL2 production upon LPS stimulation (Figure 6a,b,d). The compounds were slightly less active as inhibitors of TNF-α excretion. Compound **1** caused a statistically significant reduction in the release of this cytokine only in the two higher concentrations (1.0 and 2.5 μM, see Figure 6c). In the case of compound **2**, a statistically significant effect was observed for all tested concentrations.

## 3. Discussion

In the past five years, a series of papers on sesquiterpene lactones from *C. divaricatum* has been published by Zhang and coworkers [6,7,8,9]. They isolated and described 20 new highly oxygenated sesquiterpene lactones of germacrane type and elucidated the stereochemistry of some formerly known compounds. The germacranolides of *C. divaricatum* were divided into four structural types (I-IV). Compounds of type IV made up about 50% of the sesquiterpene lactones isolated from the plant [8,9].

Our previous work [12] described the isolation of 12-oxo-phytodienoic acid (12-OPDA) from aerial parts of *C. divaricatum*. By fractionation of a chloroform extract from the same plant material, several mixtures of sesquiterpene lactones were obtained in addition to the oxylipin. Further separation of the mixtures led to the isolation of individual compounds of different purity that were identified based on the spectral data available from the literature. Three of the compounds, all included in the structural type IV, were selected for further investigation. Compound **1,** first isolated by Goswami et al. [27] from *Inula cappa,* and compound **3,** first isolated by Gao et al. [28] from *C. triste*, were identified as constituents of *C. divaricatum* by Zhang et al. [8]. The compounds proved to be cytotoxic towards all selected cancer cell lines except for the MCF-7 cell line (resistant to compound **1**) [8] and the A459 cells (resistant to compound **3**) [7]. Cytotoxicity of **1** and **3** against normal cells had not been investigated. Compound **2** (cardivarolide G) was first described as a constituent of *C. divaricatum* by Zhang et al. [8], and its biological activity had not been evaluated previously. To assess the selectivity of the cytotoxic effect towards cancer cells, both normal and cancer cell lines were treated in vitro with different concentrations of compound **3.** The investigated cell lines were divided into three panels: prostate (normal prostate epithelial cells and two prostate cancer cells with different metastatic potential), skin (normal keratinocytes and two melanoma cell lines) and gastrointestinal (two colon cancer cell lines). Both normal and cancer cells were susceptible to compound **3**. IC_50_ values (all within a range of 8.3–35.0 μM after 24 h treatment; cell density 1.5 × 10^4^) were similar to those established earlier using HepG2 and HeLa cells (17.0 and 29.4 μM) [7]. Direct comparison of the cytotoxic effects, however, is not possible due to different cell density (2 × 10^4^) and the time of treatment (96 h) employed. Doxorubicin, used as a reference cytostatic drug, demonstrated better activity against the cell lines under investigation (IC_50_ within a range of 1.1–10.5 μM), except for the PC3 cells that were apparently resistant to the drug. Thus, the assessed germacranolide (**3**) demonstrated moderate, nonselective cytotoxic activity against the cell lines used in the experiment.

Parthenolide, a frequently investigated germacranolide with known proapoptotic activity in cancer cells, was shown to induce p53 protein—the transcription factor that controls cell cycle progression and apoptosis [31,32]. Incaspitolide A (germacranolide of type III, not known as a constituent of *C. divaricatum*) also upregulated p53 expression [14]. Some cell lines with p53 deletion proved to be resistant to doxorubicin-induced apoptosis [33]. To establish whether or not p53 plays a crucial role in the cytotoxic activity of the examined germacranolides, two osteosarcoma cell lines (with wild-type p53 status and with p53 deletion) were treated with **1** and **2** (at a concentration range of 0.014–50 µM). Our results (Figure 2 and Figure 3) demonstrated that both compounds exerted a cytotoxic effect towards the treated cells. IC_50_ values calculated for cardivarolide G (**2**) were slightly lower than those estimated for **1**. The examined lactones seem to have proapoptotic activity, as revealed by microscopic observation. Their cytotoxic effect could be observed already after 24 h treatment and did not increase significantly after another 24 h. Moreover, the cytotoxic activity was independent of the p53 status of the cancer cell line. The response of the treated cells to doxorubicin differed from the response to **1** and **2**. Doxorubicin-treated cells seem to be first cell cycle-arrested (after 24 h) and then killed (48 h), as was evidenced by a strong drop in IC_50_ value. 

Polymorphonuclear leukocytes (PMNs) play a vital role in the human immune system. The cells, among others, are involved in the regulation of the inflammatory process and immune response via the capability to produce and to respond to a variety of small proteins engaged in cell signaling (cytokines) [29,30]. Some cytokines produced by human neutrophils have proinflammatory function and are implicated in the pathogenesis of inflammatory and autoimmune diseases in humans, such as psoriasis, rheumatoid arthritis, atherosclerosis or ulcerative colitis. Certain CNS dysfunctions characterized by neuronal degradation are also regarded to be connected with neuroinflammatory processes [34]. Secretion of four proinflammatory cytokines, i.e., IL-1β (leukocytic pyrogen), TNF-α and chemokines CCL2 (monocyte chemoattractant protein MCP-1) and IL-8 (neutrophil chemotactic factor CXCL8), by LPS-stimulated human neutrophils in the absence or presence of **1** or **2** was monitored by ELISA. Both tested compounds at a concentration range of 0.5–2.5 µM did not show a significant effect on the viability of LPS-treated PMNs (Figure 4). Preincubation of human neutrophils with the examined compounds significantly and in a concentration-dependent manner diminished f-MLP-induced ROS production by the cells (Figure 5). The effect was similar to that demonstrated for 12-OPDA, another *C. divaricatum* constituent investigated earlier [12]. The germacranolides **1** and **2** significantly and dose-dependently reduced secretion of proinflammatory cytokine IL-1β (Figure 6a) and chemokines CCL2 and IL-8 (Figure 6b,d). Only at the lowest concentration of **1** (0.5 μM), a significant reduction in TNF-α secretion by LPS-stimulated neutrophils could not be achieved (Figure 6c). The effects of **1** and **2** on cytokine secretion were more pronounced than those displayed by 12-OPDA. The results suggest that *C. divaricatum* germacranolides of type IV can exert a potent anti-inflammatory effect in sub-cytotoxic concentrations. 

## 4. Materials and Methods

### 4.1. General Methods

High-resolution mass spectra were obtained in the positive ion mode using Maldi-SYNAPT G2-S HDMS (Waters Corp., Milford, MA, USA) mass spectrometer equipped with an electrospray ion source and q-TOF type mass analyzer. ^1^H NMR spectra were recorded either in CDCl_3_ or in CD_3_OD on a Bruker AVANCE III HD 400 (resonance frequency 400.17 MHz) spectrometer (Bruker Corp., Billerica, MA, USA). Optical rotation was determined in CDCl_3_ on a PolAAr31 polarimeter (Optical Activity Ltd., Huntingdon, England). RP-HPLC separations were performed using an Agilent 1200 Series HPLC system (Agilent Technologies Inc., Santa Clara, CA, USA) equipped with a column oven and a diode array detector. Analytical chromatographic separations were carried out on a Kinetex XB-C18 column (4.6 × 250 mm, 5 μm total particle size; Phenomenex, Torrance, CA, USA). Semipreparative RP-HPLC was conducted on a Synergi 4μ Fusion-RP, 80A, 250 × 10 mm column (Phenomenex), with an isocratic elution, using MeOH–H_2_O mixtures of different polarities. Conventional column chromatography was carried out on Silica gel 60 (0.063–0.2 mm, Merck, Darmstadt, Germany). TLC separations were performed using precoated plates (Silica gel 60, Art. No 5553, Merck, Darmstadt, Germany).

### 4.2. Materials

Organic solvents of analytical grade were purchased either from POCh S.A. (Gliwice, Poland) or from Merck (Darmstadt, Germany). Water was purified by a Milli-Q system (Millipore Corp., Bedford, MA, USA). MeOH and MeCN of HPLC grade were purchased from Merck. Cell culture media (except for McCoy’s 5A medium), Hanks’ balanced salt solution (HBSS), formyl-Met-Leu-phenylalanine (f-MLP), LPS (from Escherichia coli 0111:B4), propidium iodide (PI), luminol, thiazolyl blue tetrazolium bromide (MTT), 4-(2-hydroxyethyl)-1-piperazineethanesulfonic acid (HEPES) solution and L-glutamine were purchased from Sigma-Aldrich Co. (St. Louis, MO, USA). McCoy’s 5A medium was purchased from Lonza (Basel, Switzerland). Fetal bovine serum (FBS) was supplied either by Biowest (Riverside, MO, USA) or by Gibco (Grand Island, NY, USA). Phosphate-buffered saline (PBS) was purchased from Biomed (Lublin, Poland).

### 4.3. Plant Material

Seeds of *C. divaricatum* were provided by the Research Center for Medicinal Plant Resources, National Institute of Biomedical Innovation (Tsukuba, Japan). Aerial parts of *C. divaricatum* plants (grown from the seeds in the Garden of Medicinal Plants, Maj Institute of Pharmacology, Polish Academy of Sciences, Kraków) were collected in the beginning of the flowering period (August/September) and dried under shade at room temperature. A voucher specimen (3/15) was deposited in the collection kept at the Garden of Medicinal Plants, Maj Institute of Pharmacology, Kraków, Poland.

### 4.4. Isolation and Identification of Gemacranolides from Aerial Parts of C. divaricatum

Dry, pulverized shoots of C. divaricatum (267 g) were extracted four times with CHCl_3_ (1.5 L). The organic extracts were combined and concentrated in vacuo to provide 24 g of an oily residue. The residue was fractionated by conventional CC on silica gel using *n*-hexane–EtOAc gradient solvent system (up to 100% EtOAc). Collected fractions (50 mL each) were combined, as shown by TLC. Fractions 205–214, eluted with *n*-hexane–EtOAc 3:2 (*v*/*v*) were subjected to semipreparative RP-HPLC (eluent: MeOH–H_2_O mixture, 3:2, *v*/*v*; isocratic mode; flow rate: 2 mL/min) to yield 4β,8α-dihydroxy-5β-angeloyloxy-9β-(2-methylbutyryloxy)-3-oxo-germacran-6α,12-olide (**1**, 14.4 mg; see structure in Figure 1) and 4β,8α-dihydroxy-5β-angeloyloxy-9β- (3-methylbutyryloxy)-3-oxo-germacran-6α,12-olide (cardivarolide G, **2**, 61.0 mg). Moreover, 4β,8α-dihydroxy-5β,9β-di-(3-methylbutyryloxy)-3-oxo-germacran-6α,12-olide (divarolide E, 6.6 mg) and 4β,8α-dihydroxy-5β-(2-methylbutyryloxy)-9β- (3-methylbutyryloxy)-3-oxo-germacran-6α,12-olide (10.8 mg) were identified as major constituents of the fractions 205–214. Fractions 253–261, eluted with *n*-hexane–EtOAc 1:1 (*v*/*v*), were further separated by RP-HPLC (eluent: MeOH-H_2_O mixture, 7:3, *v*/*v*; isocratic mode; flow rate: 2 mL/min) to give 4β,8α-dihydroxy-5β-isobutyryloxy-9β- (3-methylbutyryloxy)-3-oxo-germacran-6α,12-olide (**3**, 9.8 mg) and a subfraction (12.5 mg) containing 2α,5-epoxy-5,10-dihydroxy-6α-angeloyloxy-9β-isobutyryloxy-germacran-8α,12-olide as a main constituent. Structures of the compounds were elucidated based on their spectral data (HRESIMS, ^1^H NMR, specific rotation) in comparison with the available literature data [7,8,27,28,35].

4β,8α-Dihydroxy-5β-angeloyloxy-9β-(2-methylbutyryloxy)-3-oxo-germacran-6α,12-olide (**1**): white amorphous powder, [α]_D_^25.7^ −41.6 (c = 0.2, CHCl_3_); UV (MeCN-H_2_O) λ_max_ 214 nm; ^1^H-NMR spectrum (400.17 MHz, CDCl_3_, see Supplementary Materials Figure S2) in accordance with literature [22]; HRESIMS (pos. mode) *m*/*z*: 503.2260 [C_25_H_36_O_9_Na]^+^; calc. 503.2257.

4β,8α-Dihydroxy-5β-angeloyloxy-9β-(3-methylbutyryloxy)-3-oxo-germacran-6α,12-olide (cardivarolide G, **2**): white needles, [α]_D_^26^ −69.3 (c = 0.1, CHCl_3_); UV (MeCN–H_2_O) λ_max_ 214 nm; ^1^H-NMR spectrum (400.17 MHz, CDCl_3_ and 600.21 MHz, CD_3_OD, see Supplementary Materials Figures S4 and S5) in accordance with literature [8]; HRESIMS (pos. mode) *m*/*z*: 503.2255 [C_25_H_36_O_9_Na]^+^; calc. 503.2257.

4β,8α-Dihydroxy-5β-isobutyryloxy-9β-(3-methylbutyryloxy)-3-oxo-germacran-6α,12-olide (**3**): white needles, [α]_D_^26.8^ −55.6 (c = 0.1, CHCl_3_); UV (MeCN–H_2_O) λ_max_ 208 nm; ^1^H-NMR spectrum (400.17 MHz, CD_3_OD, see Supplementary Materials Figure S7) in accordance with literature [23]; HRESIMS (pos. mode) *m*/*z*: 291.2249 [C_24_H_36_O_9_Na]^+^; calc. 491.2257.

### 4.5. Cytotoxicity of ***3*** against Human Normal and Cancer Cell Lines

Cytotoxic activity of **3** was tested on human cancer and normal cells, grouped in three panels: prostate, skin and gastrointestinal. The prostate panel comprised prostate cancer cell lines DU145 (ATCC HTB-81) and PC3 (ATCC CRL-1435) and prostate normal epithelial cells PNT2 (ECACC 95012613). Melanoma cell lines A375 (ATCC CRL-1619) and HTB140 (ATCC Hs 294T) together with human keratinocytes HaCaT (obtained as a kind gift of Prof. Marta Michalik, Department of Cell Biology, Jagiellonian University) were included in the skin panel. Two colon cancer cell lines, namely Caco-2 (ATCC HTB-37) and HT29 (ATCC HTB-38), constituted the gastrointestinal panel. Du145 cells were grown in a modified Eagle’s medium with low (1.0 g/L) glucose concentration; HT29, PC3 and PNT-2 cells were grown in Dulbecco’s modified Eagle’s medium (DMEM) and F12 HAM Nutrient Mixture; Caco-2 cells were grown in modified Eagle’s medium with nonessential amino acids (NEAA); and melanoma cells and keratinocytes were maintained in modified Eagle’s medium with high (4.5 g/L) glucose concentration. The culture media contained antibiotics and 10% fetal bovine serum (FBS). All cultures were maintained at 37 °C in a humidified, 5% CO_2_-containing atmosphere.

The examined compound (**3**) was diluted in the culture media from freshly made stock solution in MeOH (10 mg/mL) to the working concentrations (from 0 to 50 μg/mL). Cell viability was determined as described previously [36]. Cells suspended in the nutrient medium were transferred into 96-well microtiter plates (density 1.5 × 10^4^ per well) and preincubated for 24 h (37 °C, 5% CO_2_). Then, the culture medium was replaced with the medium containing different concentrations of the assessed compound (1–50 μg/mL). After 24 h of incubation, the viability of the cells was determined using colorimetric lactate dehydrogenase (LDH) assay in comparison to the controls to which corresponding aliquots of MeOH diluted with culture media were added. Cells grown in the medium without the tested compound were used as control I (negative), and the positive control (control II) was obtained by incubation of the cells in the medium containing 1% Triton X-100. LDH released from the damaged cells into the cell culture medium was quantified by measuring the absorbance at 490 nm using a Synergy II Biotek microplate reader. Cytotoxicities of the examined compounds were calculated as follows: ((absorbance of the tested sample−absorbance of control I)/(absorbance of control II−absorbance of control I)) × 100. Results were means of three independent measurements (± SD). Doxorubicin (Ebewe Pharma G.m.b.H., Unterach, Austria) was used as a reference cytostatic drug. The IC_50_ values were determined by plotting the percentage viability of the cells versus concentration either in linear or in logarithmic scale, and the appropriate calculations were made using either Microsoft Office Excel 2003 or AAT Bioquest website program (https://www.aatbio.com/tools/ic50-calculator, accessed on 30 June 2021), respectively.

### 4.6. Cytotoxicity Assessment of ***1*** and ***2*** against U-2 OS and SAOS-2 Cell Lines

Human osteosarcoma cell lines U-2 OS (ATCC HTB-96; wild-type p53 status) and SAOS-2 (ATCC HTB-85; p53 deletion) were cultured in McCoy’s 5A medium containing L-glutamine and supplemented with 10% FBS at 37 °C and in a humidified atmosphere with 5% CO_2_. First, 50 mM stock solutions of compounds **1** and **2** were prepared in DMSO and then diluted with DMSO to 1000×-concentrated working solutions (from 0.014 to 50 mM). After that, working solutions were diluted with culture media to the final 1× concentrations (0.014–50 μM).

For the MTT assay, the osteosarcoma cells suspended in the nutrient medium were seeded into 96-well microtiter plates (density 1.0 × 10^4^ per well) and preincubated for 24 h (37 °C, 5% CO_2_). Subsequently, the cells were treated with **1** or **2** at different concentrations (0.014–50 μM) and further cultivated for another 24 or 48 h. After that, MTT was added for 1 h at the final concentration of 500 ng/mL. The culture medium was removed and the formazan crystals were dissolved in isopropanol containing 40 mM HCl. Absorbance was measured using Tecan microplate reader at 570 nm with the reference wavelength of 650 nm. Graphs (Figure 2 and Figure 3) show data normalized to DMSO-treated controls (set as 100%). Data were analyzed using Origin software. IC_50_ values were calculated using the AAT Bioquest website program (https://www.aatbio.com/tools/ic50-calculator, accessed on 30 June 2021).

### 4.7. Anti-Inflammatory Activity of ***1*** and ***2***

#### 4.7.1. Isolation of Human Neutrophils

Peripheral venous blood was obtained from healthy human donors (18–35 years old) in the Warsaw Blood Donation Centre. Donors did not smoke or take any medications. They were clinically recognized to be healthy, and routine laboratory tests showed all values to be within the normal ranges. Neutrophils were isolated by dextran sedimentation and centrifugation in a Ficoll Hypaque gradient and then resuspended in (Ca^2+^)-free HBSS buffer or RPMI 1640 medium. Blood samples from three donors were used in each experiment.

#### 4.7.2. Cytotoxicity Measurement

Cytotoxicity was assessed by a standard flow cytometric probe using propidium iodide (PI) staining. After 24 h of incubation in the absence or presence of the tested germacranolide (at concentrations of 0.5, 1.0, 2.5 and 5.0 μM), the neutrophils (3.5 × 10^5^) were harvested and centrifuged (1500 r.p.m., 10 min, 4 °C), washed once with cold PBS and resuspended in 400 μL of PBS. A 5 μL aliquot of PI solution (50 μg/mL) was added to the cell suspension. After 15 min of incubation with PI at room temperature, cells were analyzed by BD FACSCalibur flow cytometer (BD Biosciences, San Jose, CA, USA), and 10,000 events were recorded per sample. The number of cells that displayed high permeability to PI, expressed as a percentage of PI(+) cells, was determined.

#### 4.7.3. Reactive Oxygen Species (ROS) Production by Neutrophils

ROS production was measured using luminol-dependent chemiluminescence test. A 70 μL aliquot of neutrophil suspension (3.5 × 10^5^) in (Ca^2+^)-free HBSS buffer, 50 μL of the tested compound solution and 50 μL of luminol (100 μM) were added to a well in a 96-well plate. ROS production was initiated by the addition of f-MLP (30 μL of 0.1 μg/mL solution) to obtain a total volume of 200 μL per well. Chemiluminescence changes were measured for 40 min, at 2 min intervals, in a microplate reader (37 °C). The background chemiluminescence produced by nonstimulated cells was also determined. The percentage of inhibition was calculated by comparison to the stimulated control without the tested compound, at the maximum luminescence.

#### 4.7.4. Proinflammatory Cytokine/Chemokine (IL-1β, IL-8, CCL-2 and TNFα) Production by LPS-Stimulated Neutrophils

Neutrophils (2 × 10^6^) were cultured in 24-well plates in RPMI 1640 medium with 10% FBS, 10 mM HEPES and 2 mM L-glutamine, in the presence or absence of LPS (100 ng/mL) and in the absence or presence of **1** or **2** (final concentrations of the examined germacranolide in a range of 0.5–2.5 μM), at 37 °C with 5% CO_2_. The neutrophils were harvested after 24 h and centrifuged (2000 r.p.m., 10 min, 4 °C). The amount of released cytokines was measured by enzyme-linked immunosorbent assay (ELISA) following the manufacturer’s instructions (BD Biosciences, USA). The effects on IL-8, IL-1β, CCL2 and TNF-α production by the neutrophils were calculated by comparing the percentages of the released agents to the stimulated control without the tested compound.

#### 4.7.5. Statistical Analysis

Results are expressed as the mean ± SEM of three independent experiments performed at least in duplicate. All analyses were performed using Statistica 13 software. The statistical significance of the differences between means was established by ANOVA with Dunnett’s post hoc test *p* values.

## 5. Conclusions

Sesquiterpene lactones of *C. divaricatum*, derivatives of 4,5,8,9-tetrahydroxy-3-oxo- germacran-6,12-olide, displayed moderate nonselective cytotoxic activity against the normal and cancer cell lines used in the study. The lack of selectivity makes them poor candidates for lead compounds in the development of antineoplastic drugs. The compounds in sub-cytotoxic concentrations significantly and dose-dependently reduced secretion of proinflammatory cytokines from LPS-stimulated human neutrophils. Based on the above results, traditional therapeutic use of *Carpesium* preparations, though supported by the anti-inflammatory activity of the germacranolides, should be treated with caution. 

## Figures and Tables

**Table 1:** Viability of normal and cancer cell lines treated for 24 h with 1–50 μg/mL of **3** (values are means of three measurements ± SD).

Concentration (µg/mL)	Cell Viability (%) ± SD							
	Prostate Normal and Cancer Cells			Keratinocytes and Melanoma Cells			Colon Cancer	
	PNT2	Du145	PC3	HaCaT	A375	HTB140	HT29	Caco-2
1	87.09 ± 1.79	68.42 ± 0.52	78.51 ± 1.08	86.79 ± 1.70	64.22 ± 1.13	81.37 ± 1.28	69.27 ± 1.75	52.79 ± 1.62
3	77.17 ± 1.32	56.28 ± 0.97	65.03 ± 1.46	74.50 ± 2.29	55.42 ± 1.44	70.79 ± 2.37	57.95 ± 1.66	38.59 ± 2.35
5	69.39 ± 0.97	44.83 ± 2.05	49.91 ± 2.02	64.39 ± 1.33	43.77 ± 1.69	50.92 ± 1.80	49.25 ± 2.00	15.05 ± 2.91
10	52.84 ± 2.31	37.72 ± 2.16	37.21 ± 1.27	30.51 ± 1.32	32.99 ± 2.30	21.49 ± 2.53	38.00 ± 2.29	1.96 ± 2.70
20	32.45 ± 1.84	23.62 ± 1.54	29.19 ± 1.89	0.79 ± 0.72	7.09 ± 1.78	4.10 ± 1.75	24.42 ± 1.10	0.31 ± 0.43
30	21.40 ± 0.45	12.49 ± 1.94	5.42 ± 2.25	0 ± 0	0.82 ± 0.65	0.59 ± 0.78	10.38 ± 1.09	0 ± 0
50	3.29 ± 1.27	0.62 ± 0.58	0.65 ± 0.33	0 ± 0	0 ± 0	0 ± 0	0.79 ± 0.78	0 ± 0
IC_50_ (IC_50_ µM)	16.39 ± 0.40(35.02)	5.08 ± 1.14 (10.85)	8.92 ± 1.02 (19.06)	7.95 ± 0.15 (16.99)	9.57 ± 0.87 (20.45)	6.36 ± 0.38 (13.59)	6.23 ± 0.89 (13.31)	3.88 ± 0.10 (8.29)
Doxorubicin IC_50_	1.38 ± 0.10	3.18 ± 0.10	>50	>4.68 ± 0.07	0.59 ± 0.04	>5.71 ± 0.05	1.53 ± 0.15	3.44 ± 0.10

## Data Availability

The data presented in this study are available on request from the authors.

## Supplementary Materials

The following are available online, Figures S1–S7: HRESIMS and ^1^H NMR spectra of compounds **1**–**3**.

## Sample Availability

Samples of the compounds are not available from the authors.
