# Supplementary material for: Germacranolides from Carpesium divaricatum: Some New Data on Cytotoxic and Anti-Inflammatory Activity
Source: Molecules. 2021 Jul 30;26(15):4644. doi: 10.3390/molecules26154644 (PMC8347481; doi:10.3390/molecules26154644)
Supplement: Supplementary file 1 [file molecules-26-04644-s001.zip › molecules-1300493-supplementary.pdf]

SUPPLEMENTARY MATERIAL

Germacranolides from *Carpesium divaricatum*: some new data on cytotoxic and anti-inflammatory activity

Natalia Kłeczek [1], Janusz Malarz [1], Barbara Gierlikowska [2], Łukasz Skalniak [3], Agnieszka Galanty [4], Anna K. Kiss [5] and Anna Stojakowska [1,*]

[1] Maj Institute of Pharmacology, Polish Academy of Sciences, Smętna street 12, 31-343 Kraków, Poland; kleczek@if-pan.krakow.pl (N.K); malarzj@if-pan.krakow.pl (J.M.); stoja@if-pan.krakow.pl (A.S.)

[2] Department of Laboratory Diagnostics and Clinical Immunology of Developmental Age, Medical University of Warsaw, 63a Żwirki i Wigury street, 02-091 Warsaw, Poland; barbara.gierlikowska@wum.edu.pl (B.G.)

[3] Faculty of Chemistry, Jagiellonian University, Gronostajowa street 2, 30-387 Kraków, Poland; lukasz.skalniak@uj.edu.pl (L.S)

[4] Jagiellonian University Medical College, Department of Pharmacognosy, Medyczna street 9, 30-688 Kraków, Poland; mfgalant@cyf-kr.edu.pl (A.G.)

[5] Department of Pharmacognosy and Molecular Basis of Phytotherapy, Medical University of Warsaw, 1 Banacha street, 02-097 Warsaw, Poland; akiss@wum.edu.pl (A.K.K.)

- Correspondence: stoja@if-pan.krakow.pl; Tel.: +48 126623254

Figures:

Fig. S1. HRESIMS spectrum of 4*β*,8*α*-dihydroxy- 5*β*-angeloyloxy-9*β*-(2-methylbutyryloxy)-3-oxo-germacran-6*α*,12-olide (**1**)

Fig. S2. ^{1}H NMR spectrum of **1**

Fig. S3. HRESIMS spectrum of 4*β*,8*α*-dihydroxy-5*β*-angeloyloxy-9*β*-(3-methylbutyryloxy)-3-oxo-germacran- 6*α*,12-olide (cardivarolide G, **2**)

Fig. S4. ^{1}H NMR spectrum of **2** in $CDCl_3$

Fig. S5. ^{1}H NMR spectrum of **2** in CD_3OD

Fig. S6. HRESIMS spectrum of 4*β*,8*α*-dihydroxy-5*β*-isobutyryloxy-9*β*-(3-methylbutyryloxy)-3-oxo-germacran-6*α*,12-olide (**3**)

Fig. S7. ^{1}H NMR spectrum of **3**

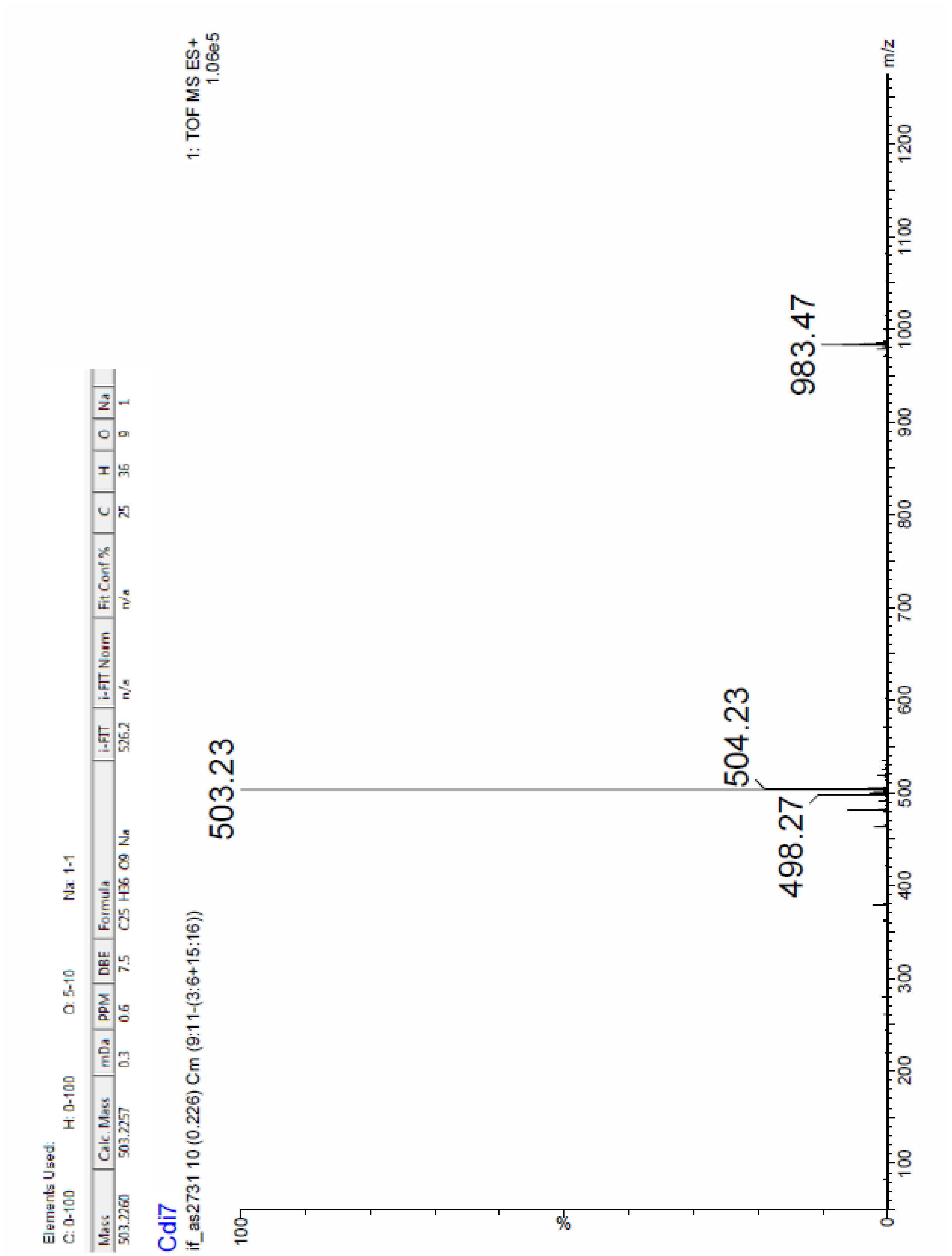

Fig. S1. HRESIMS spectrum of 4β,8α-dihydroxy- 5β-angeloyloxy-9β-(2-methylbutyryloxy)-3-oxo-germacran-6α,12-olide (**1**)

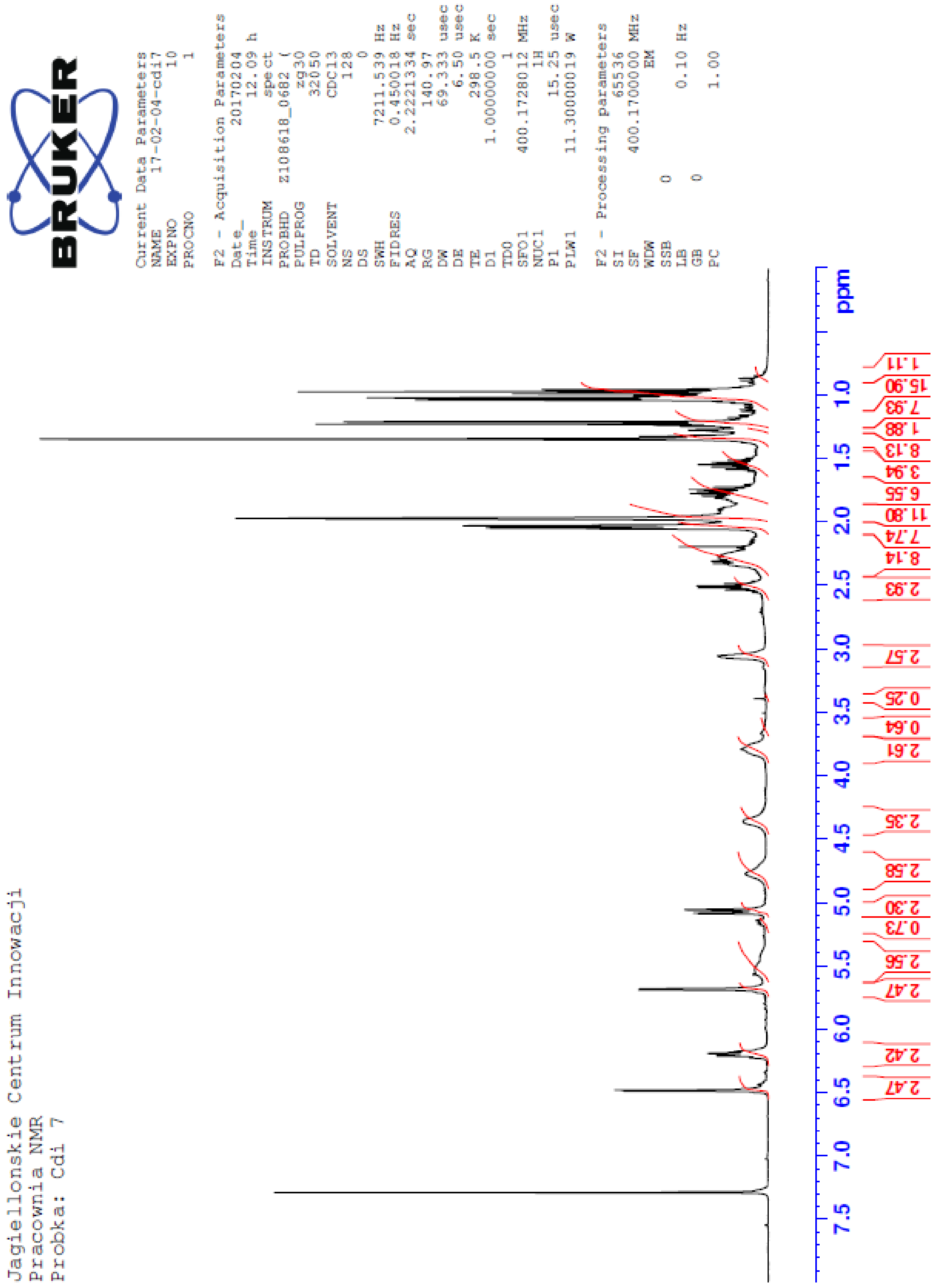

Fig. S2. ^{1}H NMR spectrum of **1**

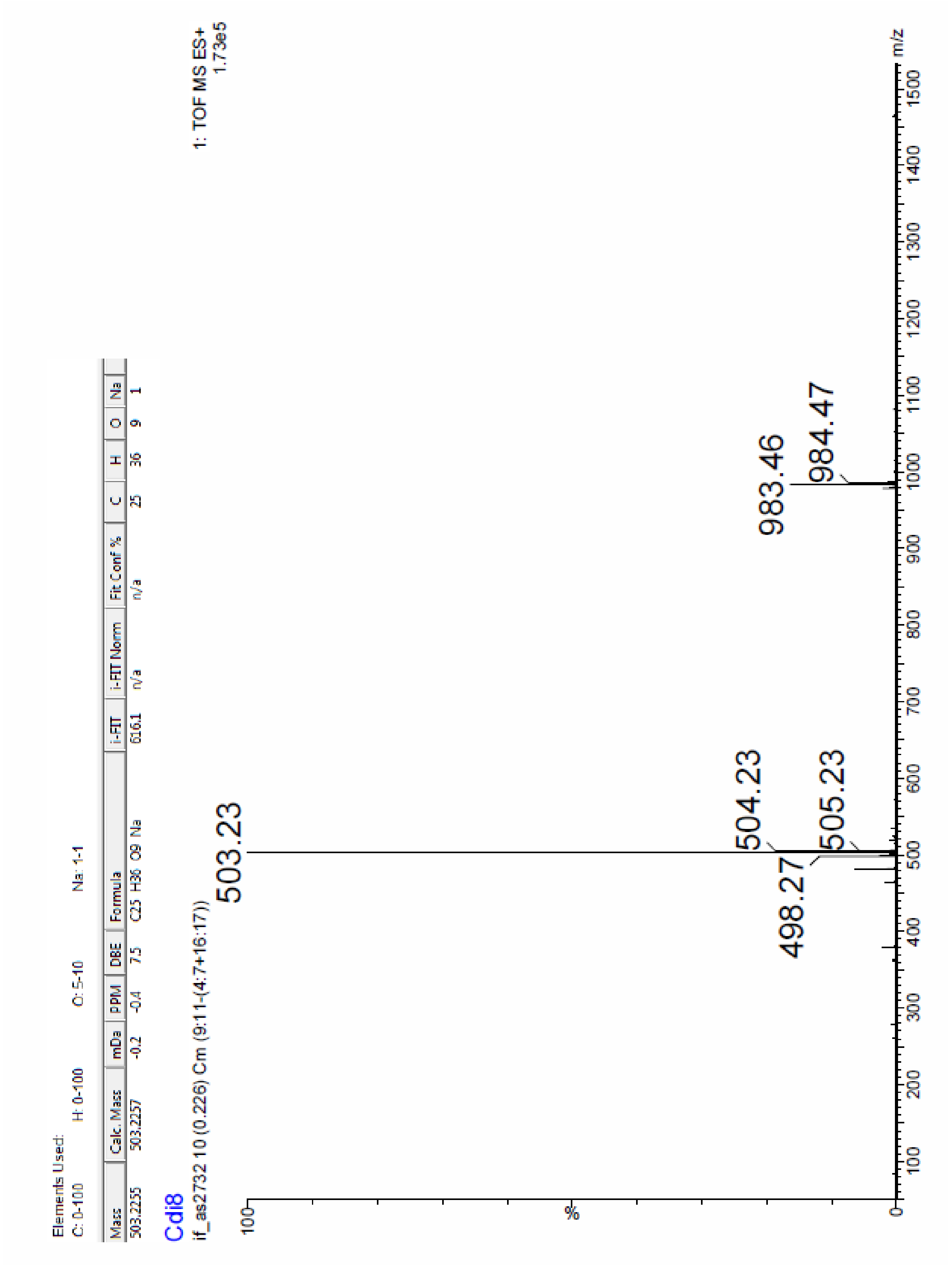

Fig. S3. HRESIMS spectrum of 4*β*,8*α*-dihydroxy-5*β*-angeloyloxy-9*β*-(3-methylbutyryloxy)-3-oxo-germacran- 6*α*,12-olide (cardivarolide G, **2**)

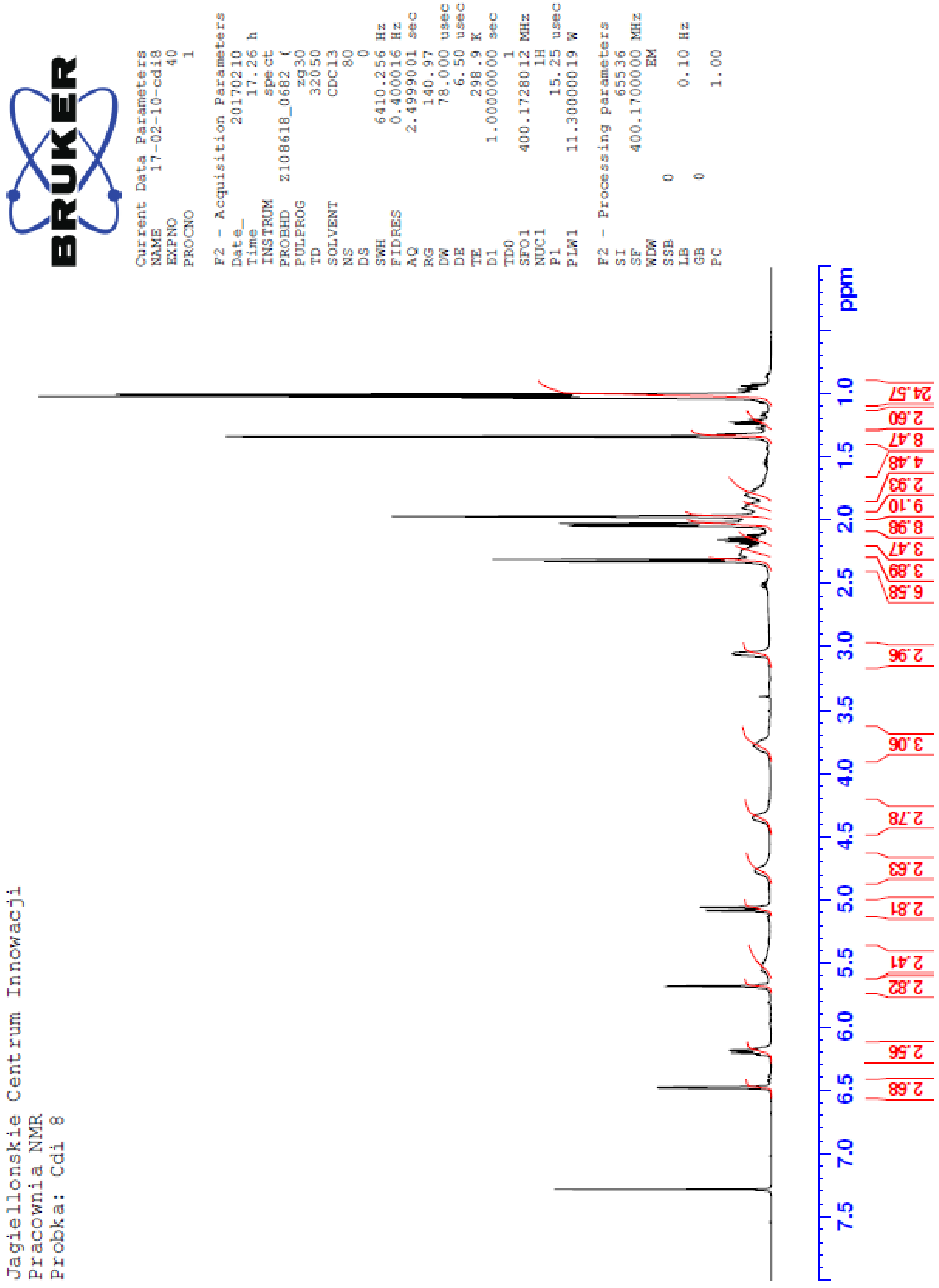

Fig. S4. ^{1}H NMR spectrum of **2** in $CDCl_3$

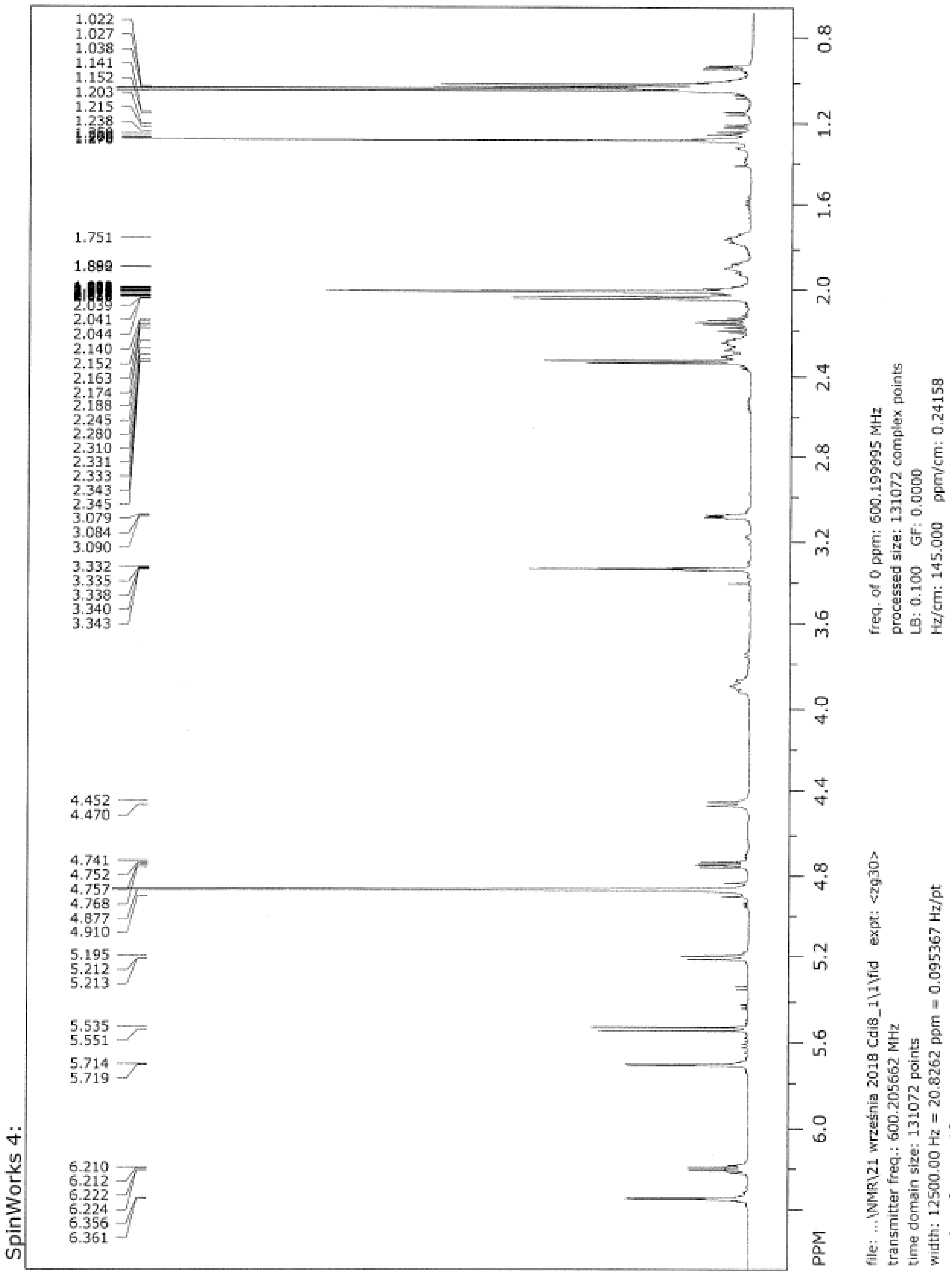

Fig. S5. ^{1}H NMR spectrum of **2** in CD_3OD

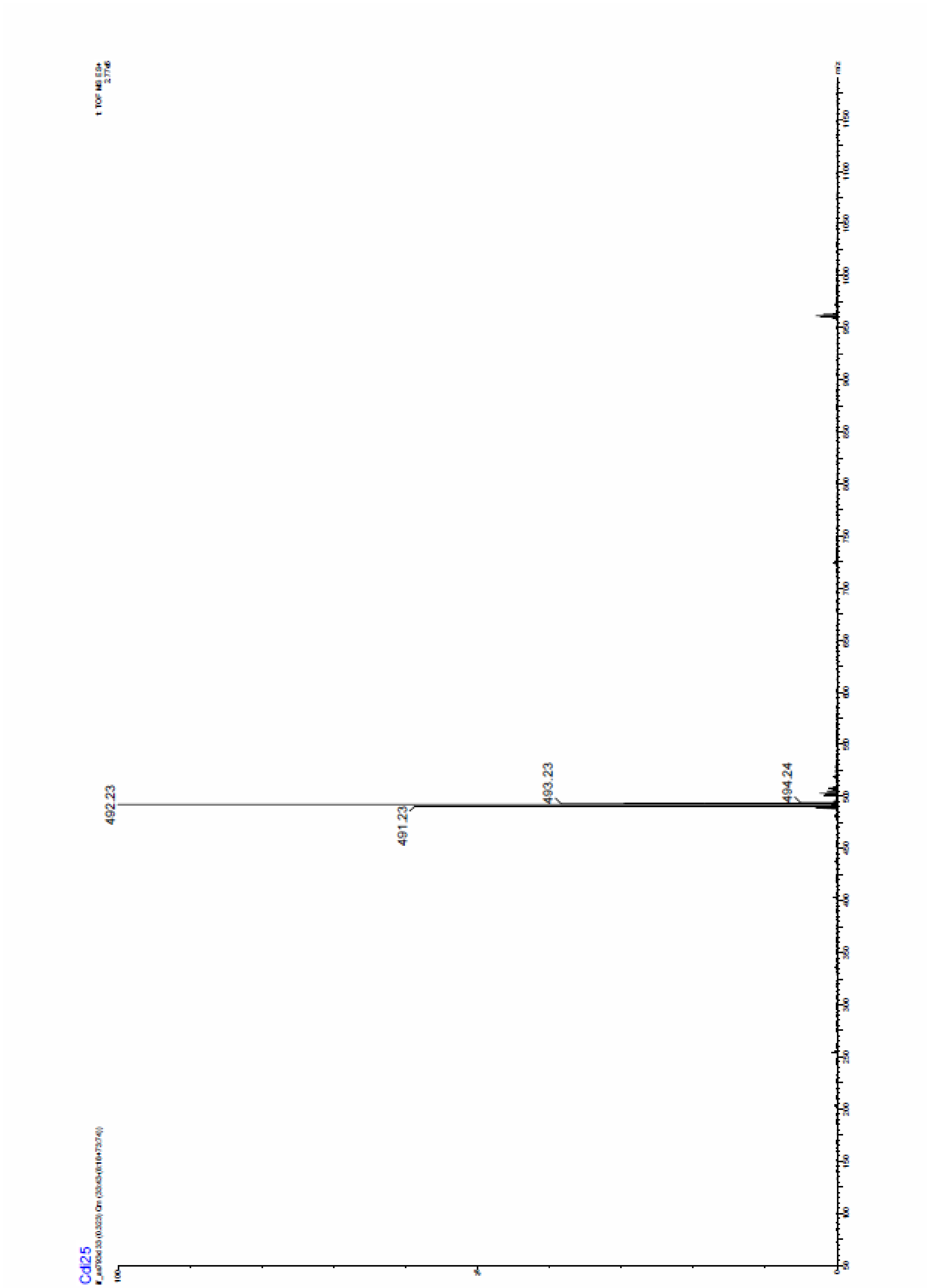

Fig. S6. HRESIMS spectrum of 4β,8α-dihydroxy-5β-isobutyryloxy-9β-(3-methylbutyryloxy)-3-oxo-germacran-6α,12-olide (**3**)

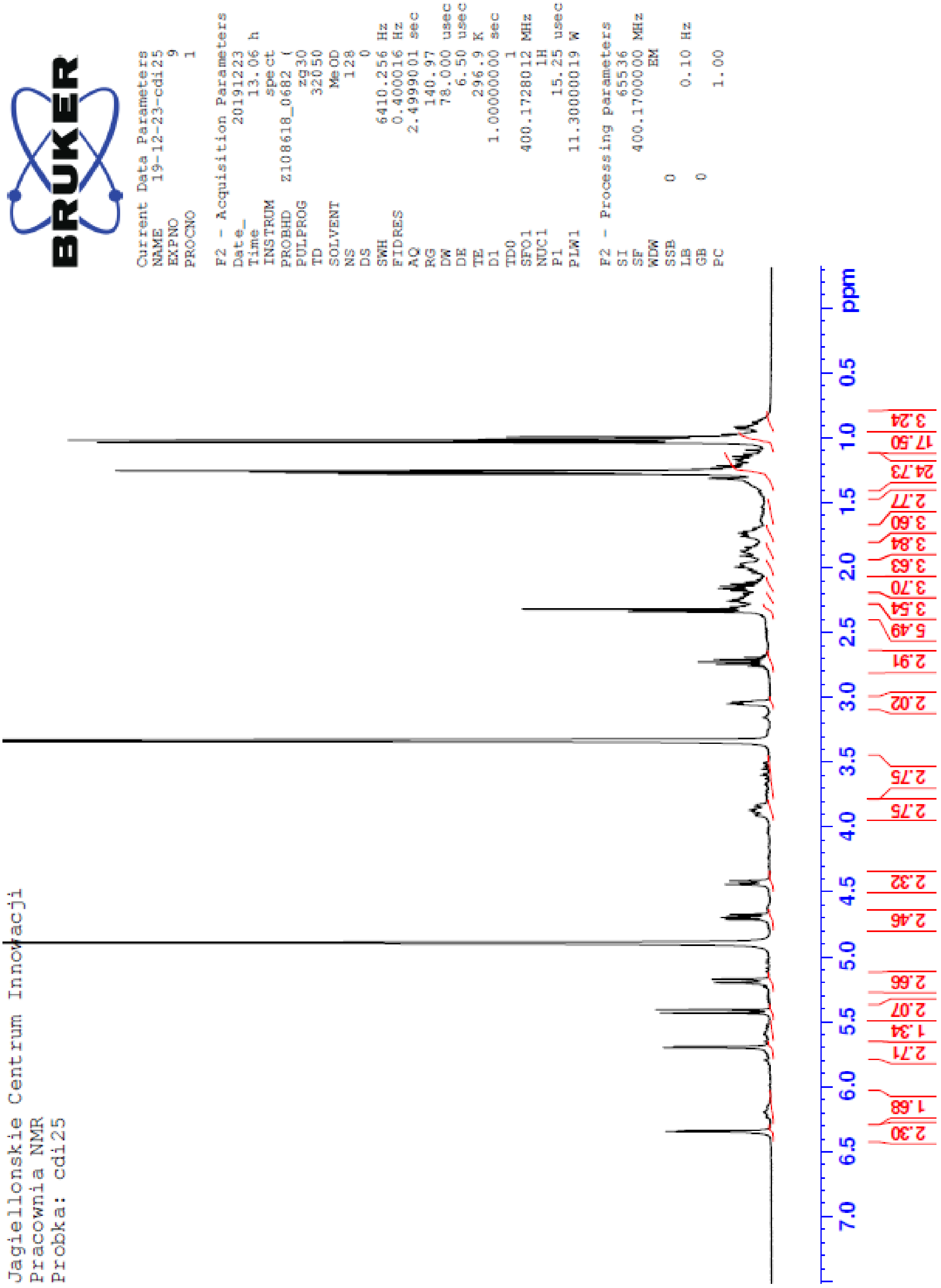

Fig. S7. ^{1}H NMR spectrum of **3**
